# Feasibility and integration of a novel bubble CPAP system into a public referral PICU in Mysuru, India

**DOI:** 10.3389/fped.2025.1685939

**Published:** 2026-01-15

**Authors:** Molly K. Rudman, Sarah Badin, Savitha M. Ramaraj, Shalini S. Rangaswamy, Paula K. Rauschendorf, Raj Prakash, Alix Boisson-Walsh, Thomas F. Burke

**Affiliations:** 1 Department of Implementation Science, Vayu Global Health Foundation, Medford, MA, United States; 2 Department of Emergency Medicine, Massachusetts General Hospital, Boston, MA, United States; 3 Department of Pediatrics, Mysore Medical College and Research Institute, Mysuru, India; 4 Department of Neonatology, King’s College Hospital NHS Foundation Trust, London, United Kingdom; 5 Maternal and Child Health Research Centre, University of Bedfordshire, Luton, United Kingdom; 6 Center for Emerging Pathogens and Infectious Diseases, Rutgers New Jersey Medical School, Newark, NJ, United States; 7 Department of Emergency Medicine, Harvard Medical School, Boston, MA, United States; 8 Department of Global Health and Population, Harvard T.H. Chan School of Public Health, Boston, MA, United States

**Keywords:** bubble CPAP, global health, non-invasive ventilation, pediatric intensive care unit, respiratory distress

## Abstract

**Objective:**

The objective of this study was to evaluate the feasibility of use and integration of a novel bubble CPAP (bCPAP) system into the PICU of the Mysore Medical College and Research Institute, India.

**Study design:**

We conducted an explanatory sequential prospective mixed-methods study using questionnaire-based surveys, focus group discussions (FGDs), and patient records. Survey and FGD participants included nurses, pediatric postgraduates, and pediatricians who worked in the PICU and used the bCPAP system. The FGDs were transcribed, coded, and systematically analyzed for emergent themes using the COM-B framework.

**Results:**

From July 31, 2023, to July 24, 2024, 81 children were treated with the bCPAP system. The median age was 6.5 months (IQR: 3–11), the median weight was 6.5 kg (IQR: 4.9–7.8), and the median treatment duration was 24 h (IQR: 18–38). Most (*n* = 72, 89%) patients treated with the bCPAP system were discharged home. Forty-eight healthcare workers completed the survey, and 29 participated in the FGDs. Survey respondents rated the bCPAP system as more effective (67%) or much more effective (17%) than previous treatments for respiratory distress. They found the integration of the bCPAP system into the PICU feasible (63%) or very feasible (35%). FGD participants reported that the bCPAP system was easy to use, portable, and required minimal training. They also noted rapid patient improvement and a reduction in the number of patients requiring mechanical ventilation.

**Conclusion:**

The bCPAP system was integrated and adopted into the PICU of this public referral facility in Mysuru, India. Further research is needed in additional settings.

## 1 Introduction

Each year, over 500,000 children under five die from lower respiratory infections worldwide (1). Twenty percent of these deaths occur in India (1). Moderate to severe respiratory distress can be treated using continuous positive airway pressure (CPAP), a form of non-invasive ventilation. Healthcare providers typically administer CPAP using a mechanical ventilator or a stand-alone CPAP machine, but these devices are often inaccessible in low- and middle-income countries (LMICs) (2).

Vayu Global Health Innovations, Public Benefit Corporation (Boston, MA, USA), designed a novel bubble CPAP (bCPAP) system for global access. Unlike other commercial CPAP devices, the novel bCPAP system uses a venturi-type air/oxygen blender and does not require electricity, compressed air, or advanced bioengineering support. While researchers have studied the bCPAP system in neonates (3–6), its use in patients older than 28 days has not yet been evaluated.

Four randomized controlled trials examined the use of bCPAP devices in children older than 28 days in low-resource settings, yielding mixed findings. A study conducted at a regional referral hospital in Bangladesh found that bCPAP significantly reduced mortality and treatment failure rates in children under five years with severe pneumonia and hypoxemia compared to low-flow oxygen therapy (7). Similarly, a cluster randomized controlled trial conducted across 12 secondary hospitals in Ethiopia showed that locally improvised bCPAP devices decreased in-hospital mortality rates and treatment failure in children aged 1–59 months with severe pneumonia and hypoxemia, compared to low-flow oxygen therapy (8).

In contrast, a trial in Malawi reported increased mortality among children aged 1–59 months with severe pneumonia and high-risk conditions (such as HIV and severe malnutrition) who received bCPAP compared to those on low-flow oxygen therapy (9). This trial, however, was conducted in a district hospital without daily physician supervision, among other significant gaps in basic care (9, 10). Additionally, a trial conducted in two community hospitals in Ghana found no reduction in two-week mortality among children ages one month to five years treated with CPAP for undifferentiated respiratory distress (11). A subgroup analysis demonstrated a mortality benefit among children under one year of age.

These mixed results highlight the need for further research on bCPAP use in children older than 28 days. A recent study emphasized that contextual factors, including healthcare infrastructure, training, caregiver involvement, and institutional practices, significantly influence the outcomes of children aged 1–59 months treated with bCPAP in LMICs (12).

In June 2023, our research team introduced the novel bCPAP system to the neonatal intensive care unit (NICU) of Cheluvamba Hospital, Mysore Medical College and Research Institute (MMC&RI), Mysuru, Karnataka, India. In July of 2023, pediatricians at MMC&RI moved three of the bCPAP systems to their pediatric intensive care unit (PICU) and expanded the use to infants and young children aged 29 days to 36 months.

The objective of this study was to evaluate the feasibility and integration of the novel bCPAP system into the MMC&RI PICU for children aged 29 days to 36 months.

## 2 Methods

### 2.1 Study design

We conducted an explanatory sequential prospective mixed-methods study, employing surveys, focus group discussions (FGDs), and reviewing patient records to gather information on the use of the bCPAP system in the PICU at Cheluvamba Hospital, MMC&RI, in Mysuru, India.

### 2.2 Setting

The study was conducted at MMC&RI, a public referral hospital located in Mysuru, Karnataka, India. Established in 1924, MMC&RI is one of the oldest medical colleges in the country. The hospital provides medical services to the city of Mysuru and the surrounding districts of Chamarajanagar, Kodagu, and Mandya. It primarily serves low- to middle-income populations who rely on government-subsidized healthcare. The institute offers undergraduate (MBBS) and postgraduate medical programs accredited by the National Medical Commission. Each year, MMC&RI enrolls 150 MBBS students and 153 postgraduate trainees. The Department of Pediatrics has 15 faculty consultants and offers a three-year postgraduate training program with 12 seats per year. MMC&RI also has an on-site nursing school that offers a four-year Bachelor of Science in Nursing (BSN) program, accommodating 100 students annually.

Cheluvamba Hospital, MMC&RI's dedicated women and children's hospital, has 421 beds, including 136 pediatric beds. In 2023, the hospital's ten-bed PICU averaged 68 admissions per month with an all-cause mortality rate of 12%. From 9:00 AM to 4:00 PM, the unit was staffed by four nurses, six pediatric postgraduates, and two pediatricians. Night coverage (4:00 PM to 9:00 AM) consisted of three nurses, three pediatric postgraduates, and one consultant pediatrician who also oversaw the NICU and emergency department.

The PICU was equipped with nine ventilators, including six Dräger Savina 300 ventilators (Drägerwerk AG & Co., Lübeck, Germany) and three Maquet Servo-I ventilators (Siemens, Munich, Germany). All ventilators had a CPAP mode. However, non-invasive interfaces, like nasal masks or prongs, were not available for use with these ventilators. Staff used the CPAP mode with an endotracheal tube (ETT-CPAP) to assess spontaneous breathing and readiness for extubation. The PICU also had two high-flow nasal cannula (HFNC) devices (Inspired, Vincent Medical Holding Ltd., Hong Kong S.A.R., China), although one device was non-operational between July and September 2023.

### 2.3 Intervention

**Figure 1:**
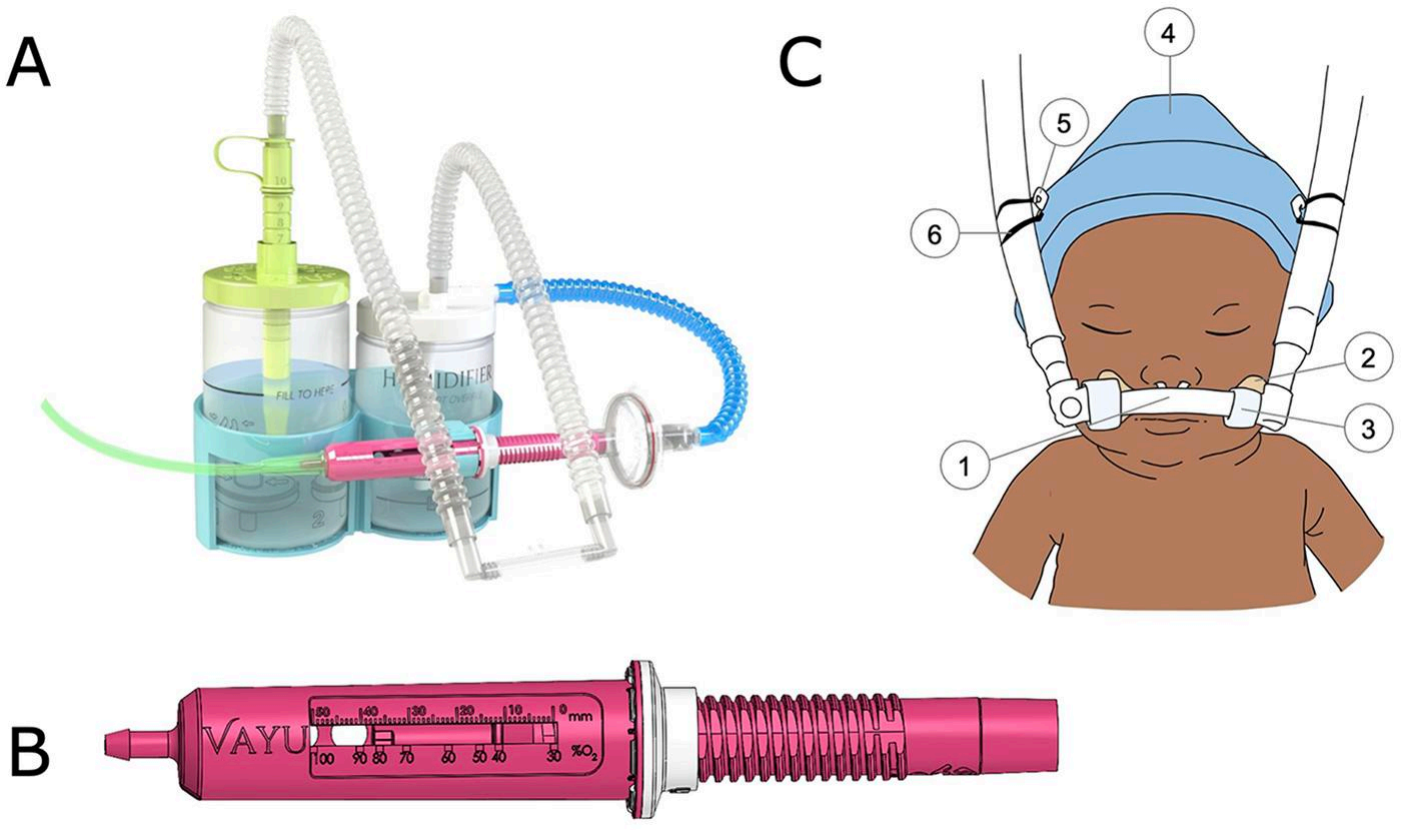
The novel bCPAP system. **(A)** Circuit setup, **(B)** venturi-type air/oxygen blender, **(C)** patient interface components: (1) silicone binasal prongs, (2) adhesive mustache, (3) soft loop fasteners, (4) hat, (5) two safety pins, (6) two rubber bands.

The novel bCPAP system (Vayu Global Health Innovations, Boston, MA, USA) administers continuous positive airway pressure (Figure 1). The system delivers flow rates of 5–10 LPM, adjustable oxygen concentrations, humidification, and pressures between 4 and 10 cm H_2_O. It uses a venturi-type air/oxygen blender and does not require electricity, compressed air, or advanced bioengineering support. The MMC&RI PICU guideline for bCPAP treatment recommended an initial pressure of 5 cm H₂O, an oxygen concentration of 30%, and a flow rate of 5 LPM. Oxygen was supplied through a reliable, hospital-wide central system, with flowmeters installed on the PICU walls. Three of the bCPAP systems were available for use exclusively in the PICU. Hospital staff reprocessed the bCPAP systems between patients.

PICU providers used the Pediatric Respiratory Severity Score (PRESS) to guide decisions on when to wean patients off bCPAP (13). If patients deteriorated while on bCPAP, care was escalated to intubation and mechanical ventilation. Bubble CPAP was often used after a period of mechanical ventilation.

The Vayu Global Health Foundation team conducted four three-hour training sessions for the NICU staff at MMC&RI from June 17 to June 19, 2023. The training covered indications, contraindications, bCPAP system assembly, patient application, monitoring, and reprocessing. Each three-hour session included a set of core lectures and one hour of hands-on practice. Initially, only medical staff who worked in the NICU or both the NICU and PICU participated in the training. All training materials, including lecture slides, were transferred to the training participants. PICU nurses were trained in August 2023 by pediatric postgraduates (“local trainers”) who had previously participated in the Vayu-led training.

### 2.4 Participants

Children admitted to the PICU between July 31, 2023, and July 24, 2024, who required non-invasive ventilation support were eligible for study enrollment. Patients were treated with either HFNC or bCPAP, with the choice of therapy determined by (1) device availability and (2) healthcare worker preference.

Survey and FGD participants included nurses, pediatric postgraduates, and consultant pediatricians who worked in the PICU and used the bCPAP system.

This study was approved by the Institutional Ethics Committee at MMC&RI (Reference: MMC EC 85/23) and is registered with the Clinical Trials Registry of India (CTRI/2024/02/063010). Pediatric postgraduates obtained written informed consent from the patient's parent or guardian prior to initiating treatment with the bCPAP system. Additionally, all healthcare workers who participated in the survey and FGDs provided written informed consent.

### 2.5 Data collection

Demographics, clinical characteristics, and treatment outcomes of patients treated with the bCPAP system in the PICU were prospectively recorded and entered into RedCap (Research Electronic Data Capture, Nashville, TN, USA) (14, 15).

The research team developed a five-point Likert-type scale survey based on a review of the existing literature. The survey was revised by U.S.-based (MKR, ABW, PKR, SB, TFB) and India-based (RP, SSR, SMR) members of the research team to ensure scientific rigor, objectivity, and cultural appropriateness. Respondents completed the survey on a tablet in a private room at MMC&RI.

An FGD guide was developed through a systematic, multi-step process with input from the U.S.-based (MKR, ABW, PKR, SB, TFB) and India-based (RP, SSR, SMR) team members. One researcher (ABW) created an initial pool of questions based on a review of relevant literature on the feasibility of bCPAP machines. The authors then refined these questions to develop an FGD guide that aligned with the research objectives. Following an analysis of the survey responses, the FGD guide was further revised to explore key themes in greater depth and address insights and gaps identified in the survey data.

Two researchers (ABW and PKR), with expertise in qualitative methods, conducted face-to-face FGDs in a private room at MMC&RI until they reached thematic saturation. The research team conducted separate FGDs for nurses and doctors to minimize potential power dynamics. FGDs with doctors were conducted in English, whereas those with nurses, who primarily spoke Kannada, were conducted in both English and Kannada. A hired translator unaffiliated with the research team facilitated the nurse FGDs. The translator had no prior experience with the bCPAP system. All FGDs were audio-recorded using a Sony ICD-UX570 digital voice recorder. English audio was transcribed verbatim by a professional transcription service (GoTranscript Inc., Harrow, United Kingdom).

### 2.6 Data analysis

#### 2.6.1 Quantitative

Demographics and clinical characteristics of patients treated with the bCPAP system were analyzed using descriptive statistics in Microsoft Excel (Microsoft Corporation, Redmond, Washington, USA). Survey data were also analyzed using descriptive statistics in Excel. A diverging stacked bar chart was created using Datawrapper (Datawrapper GmbH, Berlin, Germany) to visualize survey results (Supplementary Figure S1).

#### 2.6.2 Qualitative

The FGD transcripts were uploaded to Dedoose qualitative data analysis software (Sociocultural Research Consultants, Los Angeles, California, USA). One author (MKR) read three transcripts and developed an initial codebook using an inductive approach. Two blinded researchers (MKR, SB) independently coded each transcript using this codebook. A third researcher (ABW) reviewed and validated the coding to ensure accuracy and consistency. The three met weekly to compare and discuss their coding decisions, resolve discrepancies, address emergent themes, and refine the codebook. After each meeting, the primary coder (MKR) updated the codebook. Once the final codebook was established, the primary coder recoded earlier transcripts to ensure they were aligned with the final version of the codebook. The research team used the COM-B framework to categorize emergent themes and subthemes. U.S.-based members of the research team developed narrative summaries for each COM-B component (capability, opportunity, and motivation) and shared them with India-based team members (RP, SSR, and SMR) for feedback and modifications.

## 3 Results

### 3.1 Participant characteristics

#### 3.1.1 Clinical characteristics of patients treated with the bCPAP systems

From July 31, 2023, to July 24, 2024, 81 patients were treated with the bCPAP systems, of which 72 (89%) were discharged home and one (1%) was referred to another facility (Table 1). The majority of patients were male (*n* = 48, 59%). The median age was 6.5 months (IQR: 3–11), and the median weight was 6.5 kg (IQR: 4.9–7.8). The median duration of bCPAP therapy was 24 h (IQR: 18–38), with 79 out of 81 patients experiencing no nasal injury or pneumothorax. Following bCPAP treatment, 68 patients received low-flow oxygen therapy for a median duration of 48 h (IQR: 24–48). Children who did not improve on bCPAP (*n* = 9) were transitioned to mechanical ventilation for a median duration of 24 h (IQR: 3–48).

**Table 1:** Characteristics of children that received treatment with a bCPAP system (*n* = 81).

Characteristic	Value
Demographics	
Gender, *n* (%)	
Male	48 (59)
Female	33 (41)
Age (months), mean (SD)	7.8 (5.6)
Weight (kg), mean (SD)	6.5 (2.1)
Clinical characteristics and outcomes	
Nasal injury, *n* (%)	
No	80 (99)
Yes	1 (1)
Pneumothorax, *n* (%)	
No	80 (99)
Yes	1 (1)
Duration on bCPAP (hours), mean (SD)	32.0 (25.8)
Duration on low-flow oxygen after bCPAP therapy^a^, mean (SD)	37.6 (28.5)
Duration on mechanical ventilation after bCPAP therapy^b^, mean (SD)	31.2 (20.7)
Outcome, *n* (%)	
Discharge	72 (89)
Death	8 (10)
Referral	1 (1)

a 68 patients received low-flow oxygen therapy after bCPAP therapy.

b 9 patients received mechanical ventilation after bCPAP therapy.

#### 3.1.2 Survey and FGD participant characteristics

Forty-nine PICU medical staff participated in the study (11 nurses and 38 doctors) between April 23, 2024, and July 18, 2024 (Table 2). Five FGDs (two with nurses and three with doctors) were conducted between July 15 and July 18, 2024. Twenty-eight participants completed both the survey and an FGD, 20 completed only the survey, and one nurse participated only in an FGD.

**Table 2:** Characteristics of healthcare providers that participated in the survey and FGDs (*n* = 49).

Characteristic	All (*N* = 49)	Nurses (*n* = 11)	Doctors (*n* = 38)
Profession			
Senior pediatrician, *n* (%)	2 (4%)		
Pediatric postgraduate, *n* (%)	36 (73%)		
Nurse, *n* (%)	11 (22%)		
Participation format			
Survey and FGD, *n*	28	7	21
Survey only, *n*	20	3	17
FGD Only, *n*	1	1	0
Demographics			
Gender, *n*			
Male	11	1	10
Female	38	10	28
Age in years, mean (SD)	30.0 (6.8)	34.5 (7.8)	28.7 (6.0)
PICU experience in years, mean (SD)	2.7 (4.1)	4.0 (4.5)	2.4 (4.0)
Experience with the bCPAP system in months, mean (SD)	9.4 (2.8)	9.4 (2.9)	9.4 (2.8)
Trained by			
Vayu Global Health Trainers, *n*	25	0	25
Local Trainers, *n*	24	11	13

### 3.2 Survey quantitative results

Most survey respondents reported that they were prepared (55%) or very prepared (40%) to use the bCPAP system after receiving the training and that the training addressed relevant information about the bCPAP system well (50%) or very well (46%).

The majority of respondents reported that the bCPAP system was more effective (67%) or much more effective (17%) and easier to use (58%) or much easier to use (21%) than previous treatments for respiratory distress. Participants noted that introducing the bCPAP systems into the existing PICU infrastructure was feasible (63%) or very feasible (35%).

Participants reported that the quality of treatment for respiratory distress improved (60%) or significantly improved (33%) since the introduction of the three bCPAP systems. Healthcare providers reported that babies experienced fewer (31%) or far fewer (10%) adverse outcomes since the introduction of the bCPAP system; 31% remained neutral, and 26% reported more adverse outcomes (a finding further clarified in the Discussion).

Participants were satisfied (60%) or very satisfied (35%) with the integration of the bCPAP system into the PICU and likely (71%) or very likely (22%) to recommend it to other healthcare providers.

### 3.3 Qualitative results

The seven themes that emerged from the FGDs were categorized into the COM-B components: capability (*n* = 1), opportunity (*n* = 4), and motivation (*n* = 2) (Table 3).

**Table 3:** Themes, subthemes, and example quotes categorized by the COM-B model.

COM-B component	Theme	Subtheme	Example quote	Respondent
Capability	Training	Initial training by the Vayu Global Health team	“We were taught how to assemble [the bCPAP system] and monitor the babies. Based on the orientation class, we learned how to [apply] the [bCPAP system] to babies…[We had] a good training session…”	Doctor
		Cascade training by local champions	“We had arranged different [training] sessions for PICU nurses… We have demonstrated to them the use of [the bCPAP system]… and given one-to-one hands-on training to them…in their local language.”	Doctor
			“[We] were trained by the doctors, [pediatric postgraduates], and the training session was so good. [We] were taught everything… from basics to a higher level…”	Nurse
		Peer mentorship	'“[We] were free to ask any doubts and it was cleared by the doctors.”	Nurse
			“We told [the nurses] if they had any [questions] or troubleshooting issues, we would help them.”	Doctor
Opportunity	Factors driving the adoption of the bCPAP system in the PICU	Shortage of non-invasive ventilation options	“Noninvasive [ventilation] mechanisms were not available to us. We were directly [intubating] all babies [who needed respiratory support] and might have improved with noninvasive methods. We tried putting babies on [the bCPAP system], and [they] showed improvement. That is how we started using [the bCPAP system in the PICU].”	Doctor
		Increase in respiratory infections	“We started having a surge of respiratory cases, especially in children less than one to two years of age. For such children, we did not have a CPAP in our PICU. We only had HFNC, [low-flow oxygen therapy], ventilation, or indigenous CPAP… We thought, ‘Why not try [the bCPAP system]?'”	Doctor
		Post-extubation respiratory support	“Whenever we were weaning a case, we were [using] a ventilator-[driven] CPAP system, which was not that effective…We tried [the bCPAP system] for weaning the ventilator patients, which helped a lot.”	Doctor
		Initial success led to continued use in the PICU	“The trial of [the bCPAP system] gave us good results… We could [remove the bCPAP system] within 48 h. That made us [use the bCPAP system] more and more in the PICU…We had a good outcome for RSV-positive bronchopneumonia cases.”	Doctor
			“We had better outcomes in PICU patients when compared to the smaller babies in the NICU.”	Doctor
	Positive device features	Simple and easy to use	“The [bCPAP system] doesn't need electricity. It takes oxygen and blends it with the room air…It is very lightweight, easy to manage and install… and it's easy to learn. Compared to other CPAP [systems], [I] feel operating [the bCPAP system] is easier.”	Nurse
		Easy to learn	“[The bCPAP system] is very easy to install… [I] can teach [new staff] very easily, and they'll [learn how to use the bCPAP system] very easily, because it's a very simple and easy mechanism.”	Nurse
		Ease of monitoring	“To make sure that CPAP is working, we just look at the bubbling…In [Fisher and Paykel] machines, the pressure-generating system is lower down. We have to go [over to the machine], and look down. In the bCPAP [system]… the [pressure-generating system] is on the cot in the PICU. We can just turn around and see if it's bubbling or not. If it's continuously bubbling, it is a relief for us. The baby is breathing, and it's working well”	Doctor
		Non-electric	“[The bCPAP system does] not need electricity, that is one [advantage]…”	Nurse
		Cost-effective	“The main advantage is [the bCPAP System] is cost-effective in a resource-limited setting. In government hospitals in India and also in peripheries, because it is cost-effective, it could be implemented [more] easily than any other CPAP.”	Doctor
	Negative device features	Equipment instability	“In PICU, there is no [radiant] warmer. There's a cot, so when we put [the bCPAP system] on the bed, most of the time, it [falls] down because when the baby struggles, the whole system [falls] down on the cot, and the water spills out.”	Doctor
			“The child had pulled [on] the [bCPAP system]…The humidified water [flowed] through the [breathing] tube and…into the nostrils.”	Doctor
		Noise	“Maybe one disadvantage is because of the noise, we are not able to auscultate and get the findings of the child, the respiratory findings. Maybe in a newborn, it may not count much. A little bit older children in the PICU, we need to look at the heart sounds and then the air entry and these things.”	Doctor
			“Sometimes, when we need peace, [but the bCPAP system is making a lot of noise and], other child[ren] [are] having raised intracranial pressure, all this noise affects those babies.”	Doctor
		Displacement of patient interface	“Sometimes babies in the PICU are more vigorous than babies in the NICU, so they will move their head. They will take off [the bCPAP system].”	Doctor
	Parental involvement in patient care	Nasal prong positioning	“If the baby is very fussy, the mother will hold the nasal prongs.”	Doctor
		Monitoring	“We had better results in the PICU than in the NICU… In the PICU, parents are allowed inside to take care of [their] children…If the cap or the nasal prongs were displaced…the mothers… could easily find out that it was displaced, and we could correct the mistakes.”	Doctor
		Providing comfort	“The children…are very irritable because of hypoxia and so many things on them. If they are on the mother's lap, they are a bit more comfortable.”	Doctor
Motivation	Positive patient outcomes	Faster improvement and reduced length of hospital stay	“The cases resolved earlier, like 24 to 48 h. The patients showed earlier improvement with [the bCPAP system] than…HFNC or [ventilator-driven] CPAP… We could easily shift the patients [from the PICU to the general ward]. There was better improvement.”	Doctor
		Reduced rate of ventilation	“In the PICU, we have been able to reduce the rate of children [requiring] mechanical ventilation.”	Doctor
	Increased confidence and optimism among healthcare providers	Higher expectations for patient recovery	“[Since the implementation of the bCPAP system], we expect [better] recovery outcomes compared to our previous [experience] with HFNC. When we put a baby on [the bCPAP system], we expect that their chances of recovery are [higher]… Our confidence level has increased in noninvasive ventilation.”	Doctor

#### 3.3.1 Capability

##### 3.3.1.1 Theme 1: training

Participants described the training they received from the Vayu Global Health team as thorough and detailed. They reported that the training covered all topics adequately. One participant stated:

“We were taught how to assemble [the bCPAP system] and monitor the babies. Based on the orientation class, we learned how to [apply] the [bCPAP system] to babies…[We had] a good training session…” *Doctor*

Staff who did not attend the initial Vayu-led training received subsequent training from the pediatric postgraduates in the local language, Kannada. One participant noted:

“[We] were trained by the [pediatric postgraduates] and the training session was so good. [We] were taught everything… from basics to a higher level…” *Nurse*

Early trainees assumed informal mentorship roles by answering questions and providing guidance during the initial device use. One nurse shared:

“[We] were free to ask any doubts and it was cleared by the doctors.” *Nurse*

#### 3.3.2 Opportunity

##### 3.3.2.1 Theme 2: factors driving the adoption of the bCPAP system in the PICU

Participants reported that in July 2023 three of the bCPAP systems were transferred from the NICU to the PICU because the PICU only had one functional HFNC device for non-invasive ventilation. Several PICU doctors, who also worked in the NICU, had successfully used the bCPAP system to treat neonates. They sought to replicate the positive clinical outcomes achieved with the bCPAP system in the NICU within the PICU setting.

“We started having a surge of respiratory cases, especially in children less than one to two years of age. For such children, we did not have a CPAP [device] in our PICU. We only had one HFNC machine… and our own indigenous CPAP [a locally improvised CPAP system]… We thought, “Why not try [the bCPAP system]?”” *Doctor*

Participants also noted that the bCPAP system was helpful in weaning patients from mechanical ventilation. One doctor remarked:

“Whenever we were weaning a case, we were [using] a ventilator-[driven] CPAP system, which was not that effective…We tried [the bCPAP system] for weaning the ventilator patients, which helped a lot.” *Doctor*

Healthcare providers reported initial positive results from using the bCPAP systems in PICU patients, with many observing better outcomes in the PICU compared to the NICU. For example:

“The trial of [the bCPAP system] gave us good results… We could [remove the bCPAP system] within 48 h. That made us [use the bCPAP system] more and more in the PICU…We had a good outcome for RSV-positive bronchopneumonia cases.” *Doctor*

“We had better outcomes in PICU patients when compared to the smaller babies in the NICU.” *Doctor*

The early success helped healthcare providers gain confidence in the bCPAP system, leading to its adoption and sustained use in the PICU.

##### 3.3.2.2 Theme 3: positive device features

Participants reported that the bCPAP system was easy to use, portable, and required minimal training. One participant appreciated that the device was compatible with breastfeeding. Many participants acknowledged that the device did not require electricity.

They also mentioned that monitoring babies on the bCPAP system was more manageable than other respiratory support systems. Many participants appreciated the ability to monitor the device without standing directly at the bedside. They used bubbling in the pressure generator jar as a visual indicator of proper device function and adequate respiratory support for the patient. One participant noted:

“To make sure that CPAP is working, we just look at the bubbling…In [Fisher and Paykel] machines, the pressure-generating system is lower down. We have to go [over to the machine], and look down. In the bCPAP [device]… the [pressure-generating system] is on the cot in the PICU. We can just turn around and see if it's bubbling or not. If it's continuously bubbling, it is a relief for us. The baby is breathing, and it's working well.” *Doctor*

Additionally, many participants commented on the cost-effectiveness of the novel bPCAP system. One stated,

“The main advantage is [the bCPAP system] is cost-effective in a resource-limited setting. In government hospitals in India and also in peripheries, because it is cost-effective, it could be implemented [more] easily than any other CPAP.” *Doctor*

##### 3.3.2.3 Theme 4: negative device features

Participants reported challenges in securing the bCPAP systems to beds in the PICU. In the NICU, healthcare providers could attach the systems to the railings of radiant warmers. However, this option was unavailable in the PICU due to the absence of radiant warmers. Instead, healthcare providers placed the bCPAP system directly on the patients' beds. The soft surface of the mattress made the bCPAP system unstable and less secure. Moreover, the PICU patients tended to be more active than the NICU patients. One doctor recounted an incident where a young child pulled on the bCPAP system, causing it to tip over and spill water:

“The child had pulled [on] the [bCPAP system]…The humidified water [flowed] through the [breathing] tube and…into the nostrils.” *Doctor*

Respondents reported that the interface often became detached when the patients moved.

“Sometimes babies in the PICU are more vigorous than babies in the NICU, so they will move their head. They will take off [the bCPAP system].” *Doctor*

Respondents reported that the noise generated by the device made it difficult to auscultate heart and lung sounds. To overcome this challenge, healthcare providers disconnected the patient from the bCPAP system, performed auscultation, and then reconnected the patient. Additionally, they expressed concerns that the noise could negatively affect patients with increased intracranial pressure.

##### 3.3.2.4 Theme 5: parental involvement in patient care

The presence of parents in the PICU and their active involvement in their children's care facilitated the use of the bCPAP system in the PICU. Many healthcare providers said that if the bCPAP system became disconnected, mothers quickly noticed and alerted the healthcare team. One participant remarked:

“If the cap or the nasal prongs were displaced…the mothers… could easily find out that it was displaced, and we could correct the mistakes.” *Doctor*

#### 3.3.3 Motivation

##### 3.3.3.1 Theme 6: positive patient outcomes

Respondents reported that patients improved more quickly on the bCPAP system compared to HFNC, ventilator-driven CPAP, or improvised bCPAP. One doctor stated:

“The cases resolved earlier, like 24 to 48 h. The patients showed earlier improvement with [the bCPAP system] than…HFNC or [ventilator-driven] CPAP… We could easily shift the patients [from the PICU to the general ward]. There was better improvement.” *Doctor*

Healthcare workers observed a decrease in ventilation rates and the incidence of ventilation-acquired pneumonia with the use of the bCPAP system. They reported no complications or harm caused by the bCPAP system.

##### 3.3.3.2 Theme 7: increased confidence and optimism among healthcare providers

Many participants noted that the bCPAP system increased their confidence in the effectiveness and utility of non-invasive ventilation. They expressed that, compared to their previous experience with HFNC, they now feel hopeful about the recovery outcomes for babies receiving bCPAP therapy.

“[Since the implementation of the bCPAP system], we expect [better] recovery outcomes compared to our previous [experience] with HFNC. When we put a baby on [the bCPAP system], we expect that their chances of recovery are [higher]… Our confidence level has increased in non-invasive ventilation.” *Doctor*

## 4 Discussion

This study introduces a novel approach to CPAP research. To our knowledge, this is the first study to employ FGDs to explore bCPAP use in the PICU of a hospital in an LMIC. We gathered data from descriptive patient characteristics, surveys, and FGDs, and applied the COM-B model to identify and categorize key emergent themes. Our research was particularly innovative as it explored the use of bCPAP therapy beyond the neonatal period–an area where there remains a paucity of quality scientific evaluation.

We found that local doctors transferred three bCPAP systems from the NICU to the PICU in July 2023 in response to an increase in respiratory infections and a lack of non-invasive ventilation options in the PICU. This decision aligned with reports of increased respiratory syncytial virus (RSV) infections and hospitalizations during the 2022–2023 post-COVID-19 pandemic season (16). In the United States, there was a 70.1% rise in pediatric patients requiring advanced respiratory support compared to pre-pandemic seasons (16).

Several factors facilitated the implementation and sustained use of the bCPAP system in the PICU. Nurses and doctors reported that the device was user-friendly, required minimal training, and easily integrated into their existing workflows. Hands-on instruction, initially delivered by the Vayu Global Health team during NICU training sessions, further supported the integration of the bCPAP system into clinical practice. Staff who participated in these sessions subsequently trained additional PICU colleagues, creating a cascade training model in which early trainees formally trained successive cohorts and expanded institutional capacity. Informal mentoring also played a key role, with experienced staff answering questions, offering practical tips, and providing support to colleagues during the initial device use. This combination of structured training and peer-to-peer mentorship fostered a collaborative learning environment, promoting consistent use of the bCPAP system. Previous studies demonstrated that bCPAP systems and interfaces that are easy to use facilitate confidence among healthcare workers in their training and ability to provide care (12, 17). Additionally, training-of-trainers programs and peer mentorship were identified as key facilitators of bCPAP implementation (12, 18–20). The importance of mentorship for skill and knowledge development and maintenance in nursing practice has also been well established (21–23).

Mortality rates for infants and children treated with CPAP in low-resource settings range from 0.2% to 17% across varied levels of care, illness severity, and device type (7–9, 11). The 10% mortality rate among patients treated with the bCPAP system in the MMC&RI PICU is consistent with the facility's role as a public referral center of last resort for critically ill children from a large catchment area. Interpretation of what the 10% mortality rate in this population means is perhaps best reflected in the nurses' and doctors' reports of positive patient outcomes. Nabwera et al. found that when staff experienced good outcomes from CPAP use, their morale and confidence in the therapy improved (24). In contrast, negative outcomes reduced staff confidence and adversely affected the sustainability of CPAP use. In our study, initial positive experiences with the bCPAP system increased staff confidence in bCPAP therapy and encouraged its continued use in the PICU.

The study identified several barriers to the use of the bCPAP system in the PICU. The device was designed to attach to the railing of a radiant warmer, but since the PICU did not have radiant warmers, the devices were placed directly on patient beds. This arrangement caused the bCPAP systems to tip over and spill water occasionally. To reduce the risk of water spillage, some healthcare workers tied the device to the bed with gauze. Many staff members emphasized the need for bedside stands to support the bCPAP system properly. Future research should explore design modifications to improve the stability of bCPAP systems in the PICU.

The increased activity levels of patients in the PICU, compared to the NICU, presented challenges in administering bCPAP treatment. Patient movement often caused the hats and nasal prongs to become dislodged, an issue reported in previous bCPAP studies (7, 25). However, the presence of parents in the PICU proved to be advantageous. Mothers frequently noticed when nasal prongs were displaced and notified the healthcare team. Some mothers even assisted by holding the nasal prongs in place during therapy, underscoring the valuable role of family involvement in patient care. Other studies have shown that involving caregivers in bCPAP therapy – such as asking them to alert healthcare workers to potential danger signs, such as nasal secretions or a lack of device bubbling – can improve treatment adherence, reduce adverse events, and reduce clinician burden (3, 4, 12, 25–27).

Some healthcare workers expressed concerns about the noise generated by the bCPAP system. Providers noted that the noise made it difficult to auscultate heart and lung sounds and that the noise might be harmful to babies with increased intracranial pressure. The American Academy of Pediatrics warns that excessive noise exposure in neonates can lead to hearing loss, stress responses, and sleep disturbance (28). Research has shown that noise levels from PICU equipment often exceed the recommended 65 dBA, posing potential risks to patient well-being (28–30). Future research should examine the impact of noise generated by the bCPAP system on infant health and overall quality of care in the PICU. After the study was conducted, Vayu Global Health modified the recommended device position in the instructions for use and training materials to decrease the noise experienced by patients.

Providers reported an improvement in the quality of treatment for respiratory distress following the introduction of the bCPAP systems. They noted that patients appeared to improve more quickly on the bCPAP systems compared to HFNC or ventilator-driven CPAP. Additionally, they observed that the use of the bCPAP systems reduced the need for mechanical ventilation and decreased the incidence of mechanical ventilation-acquired pneumonia and lung injury. This finding aligns with other studies, including a meta-analysis of 21 RCTs, which demonstrated that CPAP reduces the risk of intubation in children compared to low-flow oxygen therapy (31).

Our study had several limitations. The survey was not piloted, which may have limited the research team's ability to identify and revise ambiguous or unclear questions prior to data collection. For example, one question asked, “Since the introduction of the Vayu bCPAP device, infants have experienced (fewer/more adverse outcomes),” but the term “adverse outcomes” was not defined. Thirteen of 48 respondents (27%) reported an increase in adverse outcomes. However, FGDs revealed that many interpreted “adverse outcomes” as a lack of clinical improvement during bCPAP therapy. During the FGDs, the same respondents clarified that some patients arrived in critical condition and required mechanical ventilation. As a result, their lack of improvement was more likely due to illness severity at presentation rather than the performance of the bCPAP system. Additionally, one of the FGD facilitators (PKR) participated in the June 2023 training session, which may have led some participants to offer socially desirable answers. To mitigate this bias, facilitators repeatedly emphasized openness to all feedback during the FGDs. Language barriers posed a challenge, as nurses spoke Kannada and facilitators spoke English. A translator – an MBBS intern – received one-on-one training from the research team on best practices for accurate translation. Power dynamics may have also influenced responses. To minimize this, facilitators conducted separate FGDs for doctors and nurses. However, differences in seniority within the FGDs may still have affected discussions. Some nurses worked in both the PICU and the NICU, which may have led to unintentional references to NICU experiences despite repeated reminders from facilitators to focus on the PICU experiences. Finally, generalizability of the findings is limited. The study was conducted in an urban public referral facility in India and may not reflect the perspectives or experiences of healthcare providers in other settings.

## 5 Conclusion

The novel bCPAP system was successfully adopted and integrated into the PICU of the Mysore Medical College and Research Institute in India primarily due to its ease of use and perceived effectiveness. Further study is necessary to identify optimal methods of training, mentorship, and device characteristics to support quality care and optimal outcomes across various clinical settings.

## Data Availability

The raw data supporting the conclusions of this article will be made available by the authors, without undue reservation.

## Supplementary material

Supplementary Figure S1 Survey responses from healthcare providers on the feasibility, acceptability, and usability of the novel bCPAP system.
