# Supplementary figures and images for: Feasibility and integration of a novel bubble CPAP system into a public referral PICU in Mysuru, India
Source: Front Pediatr. 2026 Jan 15;13:1685939. doi: 10.3389/fped.2025.1685939 (PMC12852426; doi:10.3389/fped.2025.1685939)

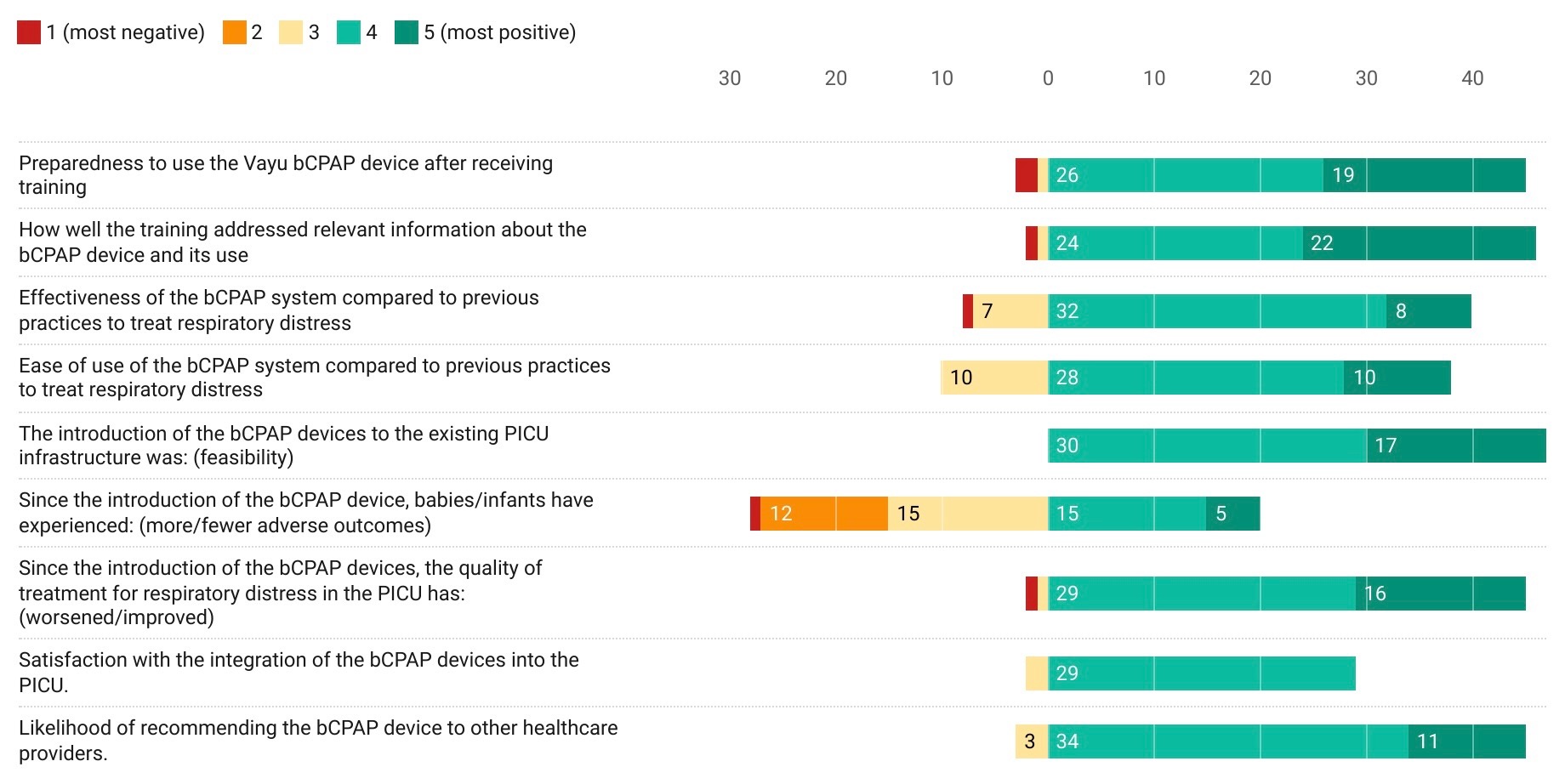

Supplement: Supplementary Figure S1 — Survey responses from healthcare providers on the feasibility, acceptability, and usability of the novel bCPAP system. [file Image1.jpeg]
